# Dr. McClelland's Rose-Pearl Base

**Published:** 1869-04

**Authors:** 


*Dr. McClelland's Rose-Pearl Base.*—For nearly two years past we have from time to time heard of Rose-Pearl, and it has been a matter of surprise that this material has not been brought directly before the profession.

At the Niagara meeting of the American Dental Association, some specimens of this work were on exhibition, and from the appearance they presented we were disposed to believe that something had at length been discovered which was at least equal to the vulcanized rubber.

In answer to a letter from us, Dr. McClelland promised to visit the Balto. College of Dental Surgery sometime during the month of February last, or, if unable to do so in person, to send an agent well qnalified to teach his method of working this material. Much to our regret neither Dr. McClelland nor his agent appeared, and consequently the little we know of this work has been gained from mere references made of it in the dental journals.

Of late there have appeared in some of the dental journals, articles for and against it, one of which is the report of a committee appointed by the "Western New York Dental Association" to investigate the fitness of Rose-Pearl as a base for artificial dentures, and which Dr. McClelland asserts, not only falsifies truth and honesty, but reflects upon his veracity.

This report condemned the Rose-Pearl in severe terms; contended that no set of it could be dried over a model without changing shape; that some sets of teeth made by Dr. McC. and worn in the mouth, and another set kept in a drawer warped to such a degree as to render them unfit for use; that owing to the shrinkage gum teeth cannot be used.

On the other hand, quite a number of practitioners, whose reliability cannot be questioned, speak of this work as giving perfect satisfaction in their practice, and assert that "as perfect fitting plates can be made with rose-pearl as it is possible to make with any other material;" that "experience warrants the assertion that it is about as easily worked, when the process is well understood, as anything else upon which artificial teeth have been mounted;" that "it is lighter than any other style of work, and plates may be made almost as thin as gold plates, and still have sufficient strength, as it is almost impossible to break the plate by bending or twisting," that "the material itself constitutes the artificial gum, so that gum teeth are not used."

We have, therefore, in this case the old saying verified, "that doctors will differ," and consequently will await further developments, before either recommending or condemning the Rose-Pearl base.

